# A public health approach to cervical cancer screening in Africa through community‐based self‐administered HPV testing and mobile treatment provision

**DOI:** 10.1002/cam4.3468

**Published:** 2020-09-23

**Authors:** Miriam Nakalembe, Philippa Makanga, Andrew Kambugu, Miriam Laker‐Oketta, Megan J. Huchko, Jeffrey Martin

**Affiliations:** ^1^ Infectious Diseases Institute‐Makerere University Kampala Uganda; ^2^ Duke University Durham NC USA; ^3^ University of California San Francisco CA USA

**Keywords:** Africa, cervical cancer screening, community based, self‐administered

## Abstract

The World Health Organization (WHO) refers to cervical cancer as a public health problem, and sub‐Saharan Africa bears the world's highest incidence. In the realm of screening, simplified WHO recommendations for low‐resource countries now present an opportunity for a public health approach to this public health problem. We evaluated the feasibility of such a public health approach to cervical cancer screening that features community‐based self‐administered HPV testing and mobile treatment provision. In two rural districts of western‐central Uganda, Village Health Team members led community mobilization for cervical cancer screening fairs in their communities, which offered self‐collection of vaginal samples for high‐risk human papillomavirus (hrHPV) testing. High‐risk human papillomavirus‐positive women were re‐contacted and referred for treatment with cryotherapy by a mobile treatment unit in their community. We also determined penetrance of the mobilization campaign message by interviewing a probability sample of adult women in study communities about the fair and their attendance. In 16 communities, 2142 women attended the health fairs; 1902 were eligible for cervical cancer screening of which 1892 (99.5%) provided a self‐collected vaginal sample. Among the 393 (21%) women with detectable hrHPV, 89% were successfully contacted about their results, of which 86% returned for treatment by a mobile treatment team. Most of the women in the community (93%) reported hearing about the fair, and among those who had heard of the fair, 68% attended. This public health approach to cervical cancer screening was feasible, effectively penetrated the communities, and was readily accepted by community women. The findings support further optimization and evaluation of this approach as a means of scaling up cervical cancer control in low‐resource settings.

## Graphical abstract

A public health approach to cervical cancer screening was feasible, effectively penetrated the communities, and was readily accepted by community women. The findings support further optimization and evaluation of this approach as a means of scaling up cervical cancer control in low‐resource settings.

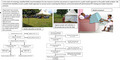

## 1 INTRODUCTION

Resource‐limited regions bear the brunt of the burden of cervical cancer incidence and mortality worldwide. In 2018, there were an estimated 528 000 incident cases and 266 000 deaths caused by cervical cancer worldwide, with 85% and 90% of these in developing countries.^1^ The situation is perhaps most dire in East Africa where age‐standardized rates reached 40 new cases of cervical cancer diagnosed and 30 deaths from the disease per 100 000 person‐years in women in 2018.^1^ This, by any definition, qualifies as a public health problem. In contrast, incidence and mortality of cervical cancer in the US are 6.4/100 000 person‐years and 1.9/100 000 person‐years, respectively.^1^ Most of this disparity is due to a lack of organized screening programs in Africa and, hence, lack of prevention of precancerous lesions becoming cancer.^2^, ^3^ For example, in Uganda, screening is erratic, opportunistic, and in some places absent translating into staggeringly low screening uptake. The prevalence of having been screened was 4.8% in one recent report from rural Uganda.^4^ While human papillomavirus (HPV) vaccination has received a significant amount of public health attention and funding as the most cost‐effective means to cervical cancer control, slow roll‐out and an exclusive focus on adolescent girls in low‐resource settings mean that screening programs will remain essential to cervical cancer prevention in these populations for the foreseeable future.

The identification of HPV infection as a causal determinant of cervical cancer and subsequent development of sensitive and specific HPV diagnostic tests have been critical technologic advances that hold promise for decreasing worldwide cervical cancer burden. One of the most impactful and innovative applications of the HPV paradigm in cervical cancer is the development of tests that have been shown to have adequate sensitivity in self‐administered vaginal specimens.^5^ In places where these tests have been investigated, such as Uganda ^6^, ^7^ and Kenya,^8^ women indeed prefer self‐administered to provider‐administered tests.^9^ Offering self‐administered tests in the community, without women having to travel to distant health facilities, should, in theory, further increase their use, and there is early evidence for this.^10^, ^11^ Regarding treatment, the World Health Organization recommends ablative therapy for a positive HPV result without further diagnostic confirmation.^12^ However, there still remain challenges in getting treatment to those with positive tests.^13^, ^14^ Women often need to travel great distances to facilities for treatment, resulting in substantial defaulting and failure to receive therapy.^11^

To address the public health problem of cervical cancer in sub‐Saharan Africa, we sought to take advantage of the technologic advancements in HPV testing and couple them with practical insights (eg, mobile treatment provision) to develop a community‐based “public health approach” to screening. We term this a public health approach because it brings services directly to the community, uses low cost processes at each step, and concedes perfection at the individual level in order to reach the greatest number of women. The approach is akin to the public health approach for HIV care and prevention ^15^—arguably one of the greatest biomedical successes of our generation. We set forth to determine the feasibility and acceptability of such a public health approach to cervical cancer screening in a demonstration project in Uganda.

## 2 MATERIALS AND METHODS

### 2.1 Overall design

We evaluated the feasibility and acceptability of a cervical cancer screening program consisting of Village Health Team Member (VHTM)‐delivered community mobilization, self‐collection of vaginal samples for HPV testing, and community‐based mobile treatment for HPV‐infected women in two rural districts of Uganda.

### 2.2 Development of community‐based message regarding cervical cancer screening

**FIGURE 1:**
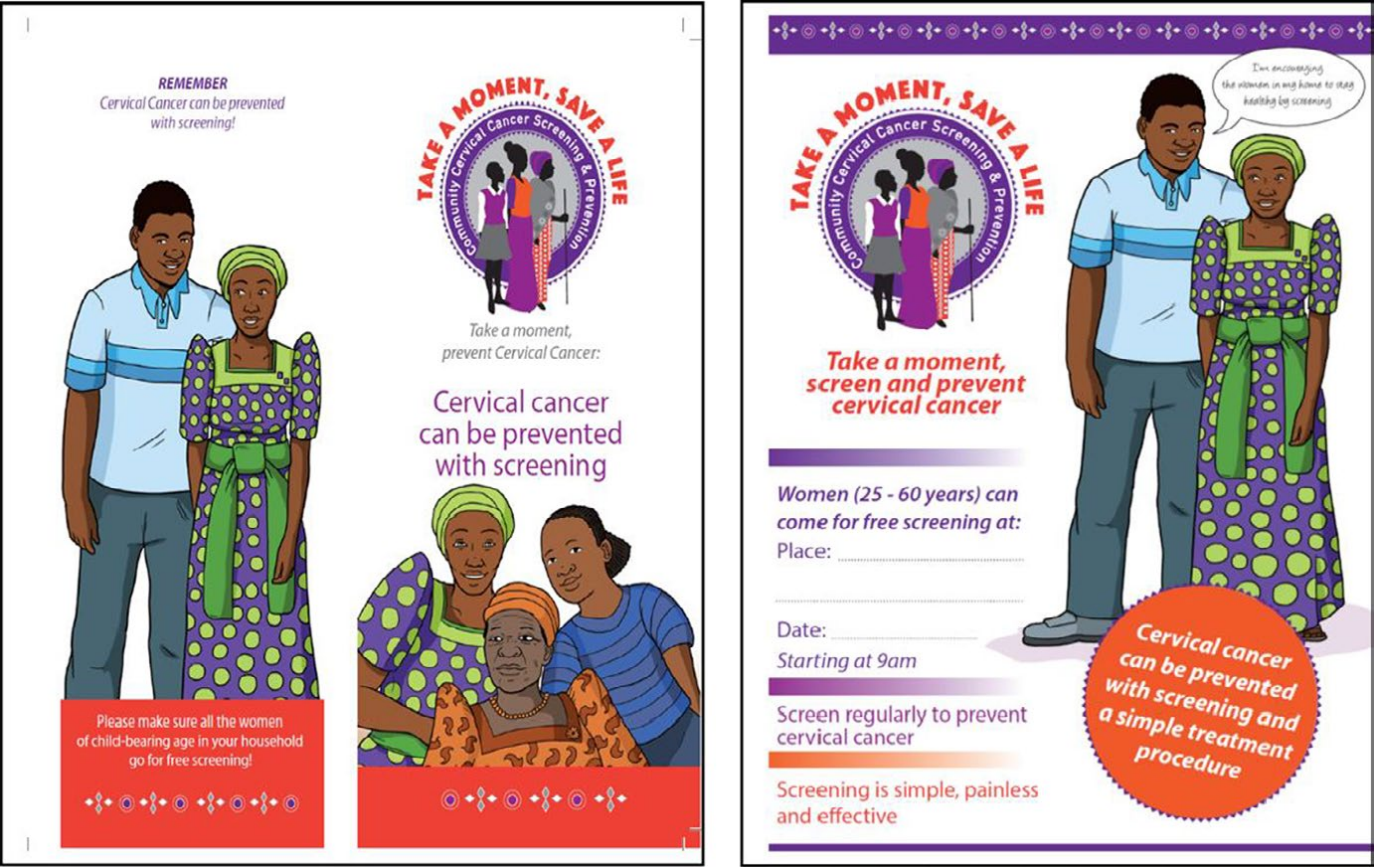
Posters used to mobilize community women to attend a health fair regarding cervical cancer screening in rural Uganda

In each district, we sought permission from district leadership to carry out cervical cancer screening and employ VHTMs. With facilitation from the district leaders, we invited key stakeholders that included the local council leaders and community elders to participate in the development of a community‐based message to encourage women to attend a health fair regarding cervical cancer screening. This process was led by Jive Media Africa, a health communication firm, who used the Information‐Motivation‐Behavioral skills model^16^ to develop posters, brochures, and short oral narratives (Figure 1).

### 2.3 Community mobilization and health fairs

#### 2.3.1 Target population and sampling

We sought to reach women 25‐60 years old residing either in Kiboga or Kyankwanzi, two rural districts with mainly agricultural economies in western‐central Uganda.^17^ The districts have a relatively higher prevalence of HIV compared to the national average (9.7% vs 7.3%).^18^ Within these districts, three subcounties were purposively selected based on the presence of Health Center Level III facility: Lwamata in Kiboga and Butemba and Ntwetwe in Kyankwanzi. In each subcounty, we chose clusters of 5‐9 parishes, which are administrative units with about 5‐20 villages and an overall population of about 1500‐6000 people. We refer to a cluster of parishes as a community.

#### 2.3.2 Community mobilization campaigns

VHTMs, also known as community health extension workers, are community members who receive training to provide basic education and care for selected health conditions. In Uganda, VHTMs are trained in various areas to provide education, linkage to care, and, in some cases, first‐line treatment at the community level.^19^, ^20^ Each village (population 300‐600 people) on average has 2‐3 VHTMs. A VHTM coordinator supervises the activities of about 8‐12 VHTMs across 4‐6 villages. VHTM coordinators in our subcounties identified 1‐2 VHTMs per village to attend a 1‐day training session, hosted by our research team, on the basics of cervical cancer, HPV self‐testing, and how to mobilize the community to attend a health fair dedicated to cervical cancer screening. We conducted a 2‐week mobilization campaign in each community, in which VHTMs informed residents about a health fair scheduled to take place within their communities offering cervical cancer screening among women aged 25‐60 years. The VHTMs spread the message to the community through placement of posters and distribution of brochures at markets and churches as well as door‐to‐door oral narratives. The campaign did not mention that the fairs were being performed under the auspices of a research study.

#### 2.3.3 Community health fairs

**FIGURE 2:**
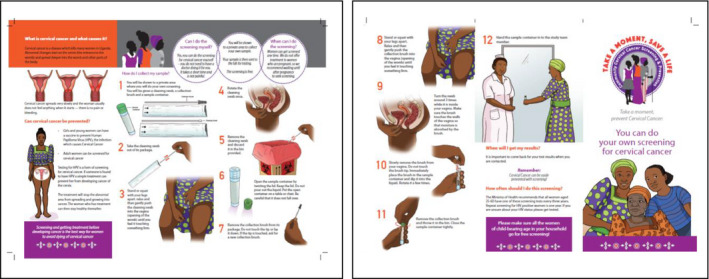
Visual aids used at the heath fair to explain cervical cancer screening in rural Uganda

We conducted 1 or 2 days of health fairs within each community. The venues for the fairs varied by community and included community centers, Health Centers, or school compounds. The VHTMs selected the venues based on their centrality within the community. Tents were assembled to provide shelter and privacy. Upon arrival at the health fair, the community women were registered by the VHTMs, working alongside the study team. Women provided their name, village of residence, age, parity, and a brief gynecological history including presence of an abnormal vaginal discharge or bleeding. The study staff provided health talks to the women in groups of 20‐30 regarding the burden of cervical cancer, risk factors, signs and symptoms, prevention by screening, and available treatment modalities for pre‐cancer stages. The sessions were interactive and facilitated with visual aids (Figure 2) and brochures.

#### 2.3.4 Self‐administered cervical cancer screening

After the educational talk, all women age 25‐60 years were asked to provide written informed consent if they wished to perform the cervical cancer screening and have their perceptions and follow‐up recorded as part of a research study. Women with a history of cervical cancer, symptoms and signs suggestive of cervical cancer (foul or pus‐like vaginal discharge or bleeding with suspicious lesion on speculum exam), or hysterectomy were excluded and referred for further evaluation as indicated. The study was approved by institutional review boards in both Uganda and the US, and all women provided written informed consent to participate. Those electing to participate listened to a demonstration on how to self‐collect a vaginal sample for the HPV test using posters and brochures. Women were provided with an unopened collection kit for HPV (Aptima^®^; Hologic/Genprobe, Inc) containing a cytobrush and a capped vial containing a collection medium. We provided floor‐length screens inside tents for privacy in which women self‐collected the vaginal specimen. Briefly, participants were instructed to place the brush inside the vagina until it met resistance and rotate it three times. The participants then placed the used brush into a collection vial with PreservCyt solution, broke the pre‐scored handle, and closed the vial prior to returning it to the health worker. From the field, the specimens were transported at ambient temperature to the Translational Laboratory at the Infectious Diseases Institute, Makerere University, Kampala Uganda. Virologic characterization of these women has been described.^21^ Finally, women were asked if they preferred to be notified of their test results by text message, phone call, home visit, or in‐person visit to the nearest health facility.

#### 2.3.5 Socioeconomic, demographic and clinical characterization and self‐collection experience

Prior to collection of the vaginal specimen, we used an interviewer‐administered questionnaire to collect demographic, clinical, and reproductive health data as well as information on accessibility of the venue. After vaginal specimen collection, women were asked to assess the experience of their participation, instructions on self‐testing, discomfort with the swab, whether they would test again, as well as if they would recommend it to someone else.

### 2.4 Result notification

Women were notified of their HPV results based on their preferred mode of notification. Text notification was considered successful if transmission of text message was confirmed (ie, phone was on, SIM card valid, line active). Phone and home visits done by the study staff were successful if participants were given their results directly by study staff. We attempted to contact the participants up to three times in a day over at least three different days within a period of 1‐2 weeks. Clinic notification was considered successful if the participant returned to her nearest clinic to access her results. Additional effort was made to notify women found to have high‐risk HPV (hrHPV) if they could not be reached based on their primary mode of notification. In case the primary mode was SMS or return to clinic, phone calls were also made. In case all this failed, the VHTMs physically visited homes and either gave results directly or made appointments for women to visit nearby clinics for their results. Women were declared lost if the VHTM confirmed that they could not find them in the community. At the time of notification, women found to have hrHPV were informed about where treatment would be available in their community and that they would be provided with transport reimbursement at the time of attendance.

### 2.5 Mobile treatment

Treatment for the HPV‐infected women was offered at a venue that was conveniently located in the women's respective communities. We typically chose existing Health Center level III or II facilities. We term this component “mobile” treatment because the treatment team brought all the supplies (eg, speculums, biopsy forceps, vinegar, as well as the cryotherapy treatment system) and moved from one community to the next. The treatment team consisted of one doctor and several nurses from the research team as well as VHTMs who assisted in the non‐clinical organization. At each site, treatment was provided for 2‐3 days, between 8 am and 3 pm. Other VHTMs carried out home visits for women who did not present for their appointment. Prior to treatment, women were asked about pregnancy status and last menstrual period, with urine pregnancy testing when indicated (MOTI test^®^; Atlas Link). Women who were found in menses were given another appointment for treatment at that facility while those found pregnant were told to follow up with their VHTM 6 weeks after delivery. Visual assessment for treatment (VAT) was done to determine appropriate treatment. Cryotherapy was used for those women with lesions covering less than 75% of the cervix, no extension to the endocervix or vagina, and no evidence of cancer. Although not required for real‐world implementation, we also, as part of the research study, performed a biopsy on all women prior to cryotherapy. Women with lesions not amenable to treatment with cryotherapy were referred for treatment at Uganda Cancer Institute (UCI) Kampala in (131 kilometers) for a Loop Electrosurgical Excision Procedure (LEEP). Women with lesions suspicious for cancer at the time of their VAT underwent biopsy. The women who were determined to have invasive cancer on biopsy were referred to the UCI.

### 2.6 Penetrance of the messaging

Four weeks after the health fair, we set out to determine the penetrance of the mobilization campaign by conducting door‐to‐door surveys within two of the communities where we had mobilized and held the fairs. We purposively selected these two communities based on connectivity to mobile internet and ability to map Global Positioning System (GPS) coordinates. Prior to the surveys, the VHTMs counted the total number of households in the selected communities. On the day of the survey, we started in the center of the communities and moved centrifugally, randomly sampling 50% of the households within a 5 km radius of the center and documenting the GPS coordinates of each. At each sampled household in which adult women were present, we asked all consenting women if they had heard about the recent health fair, how they had heard, fair attendance, and reasons for non‐attendance.

### 2.7 Statistical analysis

After determining the descriptive parameters for the population, we described the successful completion of each aspect of the screening cascade with percentages and 95% confidence intervals. We also described women's awareness of the health fairs, fair attendance, and reasons for non‐attendance with percentages and 95% confidence intervals. We evaluated various participant sociodemographic and clinical characteristics for their association with fair attendance and successful notification of HPV test results. We used risk ratios as the measure of association and used log‐binomial regression to adjust for confounding. We used a directed acyclic graph (DAG) to depict background knowledge and inform variable selection in the multivariable regression models.^22^, ^23^ All calculations were performed using Stata version 14.0 (Stata Corp.).

## 3 RESULTS

### 3.1 Community‐based screening fairs

**FIGURE 3:**
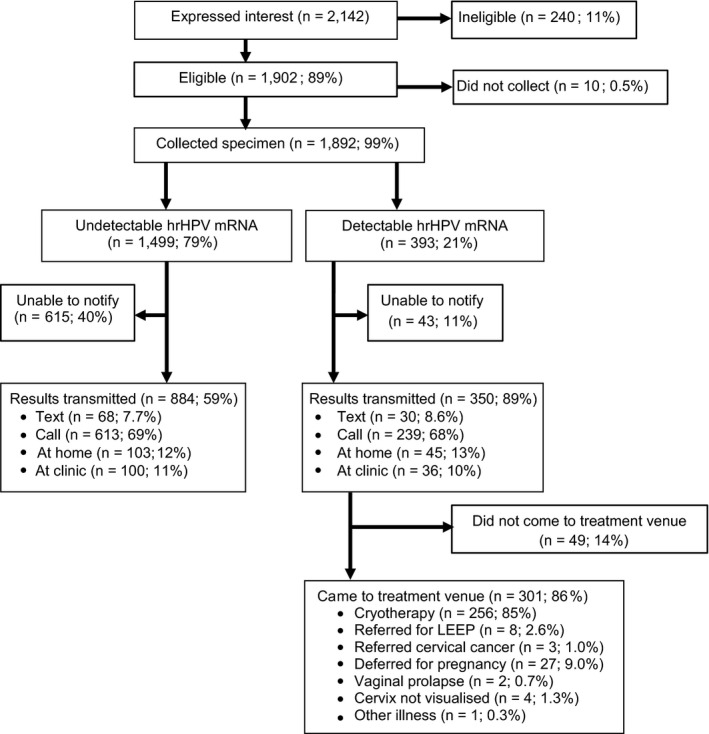
Disposition of women in rural Uganda who attended a community‐based health fair for cervical cancer screening. The fraction of women is shown who underwent screening, had their screening results transmitted to them, and who presented to a community venue for treatment. LEEP, loop electrosurgical excision procedure

Between March and November 2016, 2142 women attended one of 24 health fairs in one of 16 communities in two rural districts in western‐central Uganda and expressed interest, by virtue of registering, in being screened for cervical cancer (Figure 3). Two hundred and forty women were ineligible for screening, due to age (96%), symptoms (confirmed by pelvic exam) suggestive of cervical cancer (2.5%), or prior hysterectomy (1.3%). Of the 1902 women who were eligible and consented for cervical cancer screening, 1,892 (99%) provided a self‐collected vaginal specimen after receiving explanation. The median age of the women was 34 years (interquartile range 28‐40). The majority (79%) had only primary level education and described their job as non‐professional work (which included farming, fishing, housekeeping, and personal business). The vast majority of the women had never screened for cervical cancer (94%) including those who were HIV infected.

Regarding their perceptions of the fair, most women (95%) responded that the venue for cervical cancer screening was easily accessible within 2 kilometers of their homes and most (89%) were able to walk to the venue. The women reported to have had a positive experience with self‐collection as the vast majority of them stated that there was adequate privacy, that they would test again, and that they would recommend the testing to a friend (Table 1). In addition, most women agreed that the instructions for self‐sampling made them comfortable with the procedure.

**TABLE 1:** Opinions among women in rural Uganda regarding self‐collection of vaginal specimens at a community‐based health fair

Item	Agree	Neither agree nor disagree	Disagree
Adequate privacy for self‐collection^a^	1314 (99%)	5 (0.4%)	3 (0.2%)
Comfortable with self‐collection^b^	1059 (81%)	69 (5.3%)	177 (14%)
Can do self‐collection again^c^	1293 (98%)	11 (0.8%)	12 (0.9%)
Recommend self‐collection to a friend^d^	1298 (98%)	12 (0.9%)	9 (0.7%)

^a^ Missing in 12 participants.

^b^ Missing in 265 participants.

^c^ Missing in 31 participants.

^d^ Missing in 26 participants.

### 3.2 Results notification

The majority of the participants (77%) preferred to be contacted by phone (70% call; 7.0% text) with their HPV results; the remainder preferred coming to a clinic (12%) or a home visit by a VHTM (11%). The prevalence of any hrHPV was 21% (95% confidence interval (CI): 19% to 23%). Of the 393 women who were positive for hrHPV, 350 (89%; 95% CI: 86% to 92%) were notified, while 884 (59%; 95% CI: 56% to 61%) of the 1,499 hrHPV‐negative women were notified. Overall, 60% of the notified participants were able to be reached on the first attempt (phone call (58%), SMS (3.1%), home visit (17%), and return to clinic (21%)). In evaluating independent determinants for successful notification of results, we constructed a DAG to depict the system and inform what factors to adjust for. After relevant multivariable adjustment, we found that some education (compared to none) and preference to be notified by text (compared to via a phone call) were significantly associated with successful notification (Table 2). Having at least some secondary education had the largest direct association (adjusted risk ratio = 1.25; 95% CI: 1.08‐1.45; *P* = .004). Being employed in a non‐professional capacity (compared to being unemployed) resulted in being less likely to be notified (adjusted risk ratio = 0.86; 95% CI: 0.74‐0.99; *P* = .03).

**TABLE 2:** Evaluation of various participant sociodemographic and clinical characteristics for their association with successful notification of results from an HPV test performed at a community‐based health fair for cervical cancer screening in rural Uganda

Characteristic	No. evaluated	No. (%) successfully notified of results	Unadjusted		Adjusted	
			Risk ratio^a^ (95% CI)	*P* value	Risk ratio (95% CI)	*P* value
Age, y						
≥40	509	337 (66%)	Ref.		Ref.	
30‐39	760	506 (67%)	1.01 (0.92‐1.09)	.89	0.99 (0.89‐1.09)^b^	.80
20‐29	608	391 (64%)	0.97 (0.89‐1.05)	.51	0.97 (0.86‐1.10)^b^	.53
Marital status						
Never married	51	35 (69%)	Ref.		Ref.	
Married	1564	1025 (66%)	0.95 (0.79‐1.15)	.63	0.95 (0.77‐1.18)^c^	.66
Separated/widowed/divorced	262	173 (66%)	0.96 (0.78‐1.18)	.71	0.95 (0.75‐1.19)^c^	.64
Education						
None	305	172 (56%)	Ref.		Ref.	
At least some primary	1036	671 (65%)	1.15 (1.03‐1.28)	.012	1.19 (1.05‐1.35)^d^	.006
At least some secondary	342	237 (69%)	1.23 (1.09‐1.39)	.001	1.25 (1.08‐1.45)^d^	.004
At least some tertiary	18	12 (67%)	1.18 (0.84‐1.66)	.34	1.11 (0.76‐1.62)^d^	.56
Occupation						
Unemployed	1499	988 (66%)	Ref.		Ref.	
Employed, non‐professional	187	110 (59%)	0.89 (0.79‐1.01)	.08	0.86 (0.74‐0.99)^e^	.03
Employed, professional	191	135 (71%)	1.07 (0.97‐1.18)	.16	1.02 (0.90‐1.16)^e^	.75
Mode of notification						
Phone call	1314	852 (65%)	Ref.		Ref.	
Home visit	217	148 (68%)	1.00 (0.90‐1.11)	.96	1.01 (0.89‐1.16)^f^	.85
Return to nearby clinic	225	136 (60%)	0.98 (0.85‐1.12)	.76	0.98 (0.86‐1.11)^f^	.73
SMS^g^	121	98 (81%)	1.20 (1.04‐1.37)	.10	1.24 (1.12‐1.38)^f^	<.001
Pregnant						
Not pregnant	1687	1098 (65%)	Ref.		Ref.	
Pregnant	190	135 (71%)	1.09 (0.99‐1.20)	.07	1.06 (0.95‐1.19)^h^	.29
Parity						
0‐3	705	457 (65%)	Ref.		Ref.	
4‐6	746	485 (65%)	1.00 (0.93‐1.08)	.94	1.04 (0.94‐1.14)^i^	.45
>6	426	291 (68%)	1.05 (0.97‐1.15)	.22	1.05 (0.93‐1.20)^i^	.42
Prior cervical cancer screening						
Never screened	1791	1176 (66%)	Ref.		Ref.	
Screened	86	57 (66%)	1.01 (0.86‐1.18)	.91	0.91 (0.75‐1.12)^j^	.38
HIV infection, via self‐report						
HIV uninfected	1498	986 (66%)	Ref.		Ref.	
HIV infected	158	99 (63%)	0.95 (0.84‐1.08)	.44	1.00 (0.87‐1.14)^k^	.99

^a^ Risk ratio depicts the probability of successful notification in the index group divided by the probability of successful notification in the reference group.

^b^ Adjusted for education, HIV infection status, marital status, notification mode, occupation, parity, pregnancy, and prior cervical cancer screening. Age and notification mode missing in 15 participants.

^c^ Adjusted for age, education, HIV infection status, notification mode, occupation, parity, and pregnancy. Marital status and notification mode missing in 15 participants.

^d^ Adjusted for age, HIV infection status, marital status, notification mode, occupation, parity, pregnancy, and prior cervical cancer screening. Education and notification mode missing in 191 participants.

^e^ Adjusted for age, education, HIV infection status, marital status, notification mode, parity, and pregnancy. Occupation and notification mode missing in 15 participants.

^f^ Adjusted for age, education, HIV infection status, marital status, occupation, and pregnancy. Notification mode missing in 15 participants

^g^ Denotes short messaging service, commonly known as text messaging.

^h^ Adjusted for age, education, HIV infection status, marital status, notification mode, occupation, and parity. Pregnancy and notification mode missing in 15 participants.

^i^ Adjusted for age, education, HIV infection status, marital status, occupation, and pregnancy. Parity and notification mode missing in 15 participants.

^j^ Adjusted for age, education, and HIV infection status. Prior cervical cancer screening and notification mode missing in 15 participants.

^k^ Adjusted for age, education, marital status, notification mode, occupation, parity, pregnancy, and prior cervical cancer screening. HIV infection, self‐reported, and notification mode missing in 236 participants.

### 3.3 Mobile treatment

Of the 350 HPV‐positive women who were notified and asked to return for treatment, 301 (86%; 95% CI 82%‐89%) returned for treatment at the mobile venue in their community (Figure 3). The vast majority of the women (85%) were eligible for immediate biopsy and cryotherapy, while others were deferred for various reasons that included pregnancy (9.0%), vaginal prolapse (0.7%), inability to safely administer cryotherapy due to cervical position (1.3%), referral to Mulago Hospital for LEEP or suspicion for invasive cancer (3.6%), or concurrent systemic illness (0.3%). Of the 8 women who were referred to Mulago for LEEP, 5 of them complied and received treatment, while 2 of the 3 patients with invasive cancer were able to comply and receive treatment. All three women who were found to have cervical cancer were contacted and referred for treatment.

### 3.4 Penetrance of the messaging

**FIGURE 4:**
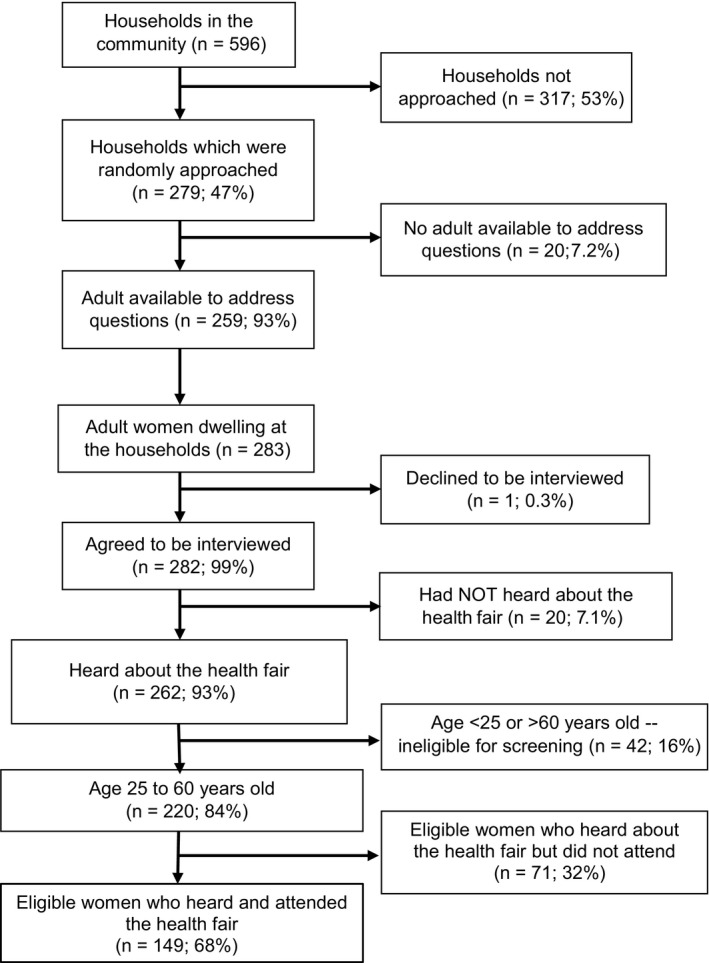
Penetrance of the mobilization message in the communities in which the health campaigns took place in rural Uganda. Penetrance was determined using a randomly selected sample of households within a 5 km radius of the center of the community. At each selected household in which there was an adult available to address our questions, we invited all women aged 18 or over to participate in a survey

After the fairs were completed, VHTMs counted all of the households within a 5 kilometer radius of the community center in two of the communities. We then set forth to randomly survey 50% of these households. Of the 596 households identified by the VHTMs in these two communities, a total of 279 (47%) were approached on the days of the survey, at which 259 (93%) had an adult available to address questions (Figure 4). At these residences, we identified 283 adult women within, of whom 282 (99%) agreed to interview. Most women (93%; 95% CI: 89%‐96%) reported hearing about the fairs. Direct communication from the VHTMs was most common means of hearing the message (68%), followed by hearing from a neighbor (27%). Of those who had heard of the fairs and were eligible, 68% (95% CI: 62%‐74%) attended. Reasons for non‐attendance included intention to attend a future fair (64%), too busy (21%), distance (6.0%), irrelevance (3.0%), fear (5.0%), and partner refusal (0.9%). Only being married (adjusted risk ratio = 1.55; 95% CI: 1.05‐2.30; *P* = .028) was associated with fair attendance but age, occupation, educational level, and parity were not (Table 3).

**TABLE 3:** Evaluation of various participant sociodemographic and clinical characteristics for their association with fair attendance in a community‐based campaign for cervical cancer screening in rural Uganda

Characteristic	No. evaluated	No. (%) attending the fair	Unadjusted		Adjusted	
			Risk ratio^a^ (95% CI)	*P* value	Risk ratio (95% CI)	*P* value
Age, in years						
20‐29	76	58 (76%)	Ref.		Ref.	
30‐39	55	34 (62%)	0.81 (0.64‐1.03)	.089	0.83 (0.63‐1.10)^b^	.24
≥40	89	57 (64%)	0.84 (0.69‐1.02)	.086	0.88 (0.69‐1.13)^b^	.32
Marital status						
Never married	31	14 (45%)	Ref.		Ref.	
Married	147	108 (73%)	1.62 (1.09‐2.42)	.017	1.55 (1.05‐2.30)^c^	.028
Separated/widowed/divorced	42	27 (64%)	1.42 (0.91‐2.23)	.12	1.41 (0.91‐2.29)^c^	.12
Education						
None	23	11 (48%)	Ref.		Ref.	
At least some primary	146	101 (69%)	1.45 (0.93‐2.25)	.10	1.26 (0.82‐1.94)^d^	.29
At least some secondary	50	37 (74%)	1.52 (0.96‐2.40)	.76	1.35 (0.86‐2.12)^d^	.19
Occupation						
Unemployed	10	5 (50%)	Ref.		Ref.	
Employed, non‐professional	192	128 (67%)	1.33 (0.71‐2.50)	.37	1.21 (0.67‐2.19)^e^	.53
Employed, professional	17	15 (88%)	1.76 (0.93‐3.36)	.084	1.50 (0.81‐2.78)^e^	.19
Parity						
0‐3	88	60 (68%)	Ref.		Ref.	
≥4	132	89 (67%)	0.93 (0.78‐1.1)	.45	1.07 (0.85‐1.35)^f^	.56

^a^ Risk ratio depicts the probability of fair attendance in the index group divided by the probability of fair attendance in the reference group.

^b^ Adjusted for education, marital status, occupation, and parity.

^c^ Adjusted for age, education, occupation, and parity.

^d^ Adjusted for age, marital status, occupation, and parity.

^e^ Adjusted for age, education, marital status, and parity.

^f^ Adjusted for age, education, marital status, and occupation.

## 4 DISCUSSION

In a population which had a very low prevalence of prior cervical cancer screening, we implemented a community‐based approach to screening featuring VHTM‐delivered community mobilization, self‐collected HPV testing, and mobile treatment. This is one of the first demonstrations of this package of interventions, all performed where women live in their communities. We found that a VHTM‐delivered mobilization effectively penetrated communities and that there was great interest in screening, acceptability of self‐collection of vaginal specimens, ability to notify women of their results, and good compliance with treatment among those who were HPV infected. Finally, we found that several socioeconomic characteristis (eg, education) were associated with greater engagement in the process, providing clues as to how further research may seek to optimize the approach.

Our program featured community‐based screening, VHTMs, and self‐administered specimen collection. Although WHO recommends protocols that radically simplify the procedures and technologies for screening and treatment, there are additional inherent barriers within resource‐constrained settings that must be addressed for effective implementation.^24^ Among women who are willing or able go to health care facilities, it is difficult for all facilities to maintain the supplies and trained personnel for counseling and screening. Further still, services may not be offered at that particular time or day when the woman is able to come to the clinic or long waiting time may all increase cost to individuals. Additionally, clinics where screening is offered are often far from a woman's home and this may limit access due to transportation challenges. Even the facilities that are most well resourced and well attended in Uganda, such as HIV‐care facilities, struggle to provide screening to their population. Despite the WHO recommendation to integrate cervical cancer screening into existing HIV care services,^25^ most of our HIV‐infected women had never been screened, despite the majority being in care. We choose to screen through community health fairs because of the inherent efficiences of high volume services and the convenience it provides to women, a belief that has been borne out in several studies comparing community‐based screening to health facilities.^26^, ^27^, ^28^

In our approach, we used VHTMs within their communities, similar to other studies who used the community health work force within their communities.^26^, ^28^ Our results show that the VHTMs were able to effectively penetrate the community with the mobilization message and motivate a high percentage of women to attend the fair. Once at the fair, almost all the women were able to provide a vaginal sample. Indeed, other evidence shows that self‐collection of vaginal specimens is very acceptable^29^ and increases screening uptake,^7^, ^10^ which ultimately increases detection of disease at the population level, offsetting the slight decrement in sensitivity associated with self‐collection at the individual level.^30^

When provided the choice of receiving results through a home visit, clinic visit, phone call, or text, the majority of the women preferred to receive results via a phone‐based method. This is similar to that of a Kenyan study where 71% of the participants preferred receiving their results by phone communication (call or text).^11^ Indeed, evidence has proved that mobile phones have become the most accessible form of communication, providing information that results in improved health outcomes and/or changed health behaviors.^31^ Overall, across all notification methods, almost all of those who tested hrHPV positive received their results (89%). By design, we made more attempts to reach the HPV‐infected women, and we were able to reach more of them in actual practice, proof of concept that a large fraction can be reached. The only other available study in Uganda that utilized community‐based self‐collection for HPV testing reported reaching just 47% of their HPV‐infected women by phone.^14^ This study was done in an impoverished urban community, which may have contributed to limited communication via the phone.

To date, there is no prior report of mobile treatment offered in a campaign style for cervical cancer. In our study, most of the HPV‐positive women (86%) were able to come to the treatment venue within their community. The women were facilitated with transport reimbursement in addition to VHTMs searching for them if they failed to make their appointment. Our study registered a slightly higher return for treatment compared to a similar study in Uganda,^10^ which also offered transport reimbursement but, in contrast to our work, held their treatment at larger facilities that were on average likely farther away from participants. Other studies where women were referred to hospitals with no reimbursement for transport have even worse return: 39%^28^ within East Africa, and (59%)^32^ and (54%)^33^ outside the East Africa. Our approach is novel because it brings treatment close to the patient through a mobile treatment team that overcomes the cost and logistical barriers to implementing and maintaining treatment capacity in all local health facilities. Our approach recognizes that it will never be cost‐efficient to staff a large number of clinics in order for most/all women to have a treatment‐equipped clinic near them. Moreover, because ablative treatment cannot be considered urgent, all clinics do not have to have it. Instead, our approach creates a very small team of expert personnel who can efficiently move across a region. Furthermore, having dedicated treatment days for a large and predictable number of known HPV‐positive patients will optimize cost‐efficiency compared to basal low‐level screening which yields an erratic number of women who need treatment.

There are trade‐offs in our public health approach. Indeed, we concede that our approach prioritizes greater community coverage over optimal care for each individual. First, women are told about the need for screening through mass communication as opposed to an individual‐level letter, phone call, or communication from a personal physician, as would happen in a resource‐rich setting. Second, self‐collection of a vaginal specimen is less sensitive than what could be achieved with clinician‐collected cervical specimen. However, we and others have recogized that self‐collected vaginal sampling has higher acceptability, hence, potentially reaching more women.^5^, ^7^, ^8^, ^9^ Third, we did not expend much resources on notifying the HPV‐negative women, who may be left with the stress of uncertainty concerning their results. Fourth, we treated all HPV‐infected women, which will certainly overtreat many, as opposed to using colposcopy and biopsy to guide treatment. Fifth, without a biopsy, our approach could miss some cancer and, hence, undertreating some women by just giving cryotherapy. However, conceding perfection at the individual level is not novel and, in fact, underlies the entire screen and treat philosophy which has been endorsed by the WHO and ASCO for low‐resource settings.^12^, ^34^ Therefore, we believe this approach is justified because it allows for a limited amount of resources to reach a larger number of women. Furthermore, what is novel about our approach is that places all of the interventions in one package that is more convenient for women. Generally, this is similar to the WHO public health approach to HIV treatment and care ^15^ where antiretroviral therapy was initiated without plasma HIV RNA testing, resistance testing, or attention to bringing back patients who are lost to follow‐up. Some enhancements like same‐day point‐of‐care HPV testing and treatment will further improve this public health approach to cervical cancer screening. Finally, we can envison how HPV vaccination for children could be added to the approach in order to achieve a comprehensive strategy for cervical cancer elimination.

Our study had several limitations. The most prominent is that our public health approach was evaluated in the context of a research study. Although the initial mobilization did not mention research, once women arrived at the health fairs they became aware that our demonstration project was part of a research study. This may have become known to other women which then may have discouraged their choice to come to later fairs. On the other hand, that we were able to reimburse for transportation among those HPV‐infected women who returned for treatment may have increased compliance with treatment as compared to a setting in which reimbursement is not possible. Another limitation is in our assessment of the penetrance of our message within the respective communities. Because of limited resources, we were forced to limit our survey ascertainment to a 5 km radius of the community center. Because residents at this radius had nearly uninformly heard about our fairs, we were not able to determine at what radius penetrance begins to fade. While this is not a threat to the validity of any of our findings, it does limit our understanding of the true penetrance of our campaign in the community.

In conclusion, the public health problem of cervical cancer requires public health solutions. We have shown that a VHTM‐led community‐based campaign for cervical cancer screening was feasible, effectively penetrated communities, and was readily accepted by community women with good retention through to treatment. The findings support further optimization and evaluation of this approach as a means of scaling up cervical cancer control in low‐resource settings. Our approach can be implemented now and should be considered by policy makers for wider implementation to ultimately reduce the burden of cervical cancer in their regions. It also merits further research by optimizing penetrance, overcoming whatever barriers might exist for some women, and optimizing cost‐efficiency at every step.

## CONFLICT OF INTEREST

The authors declare that they have no competing interests. The funders had no role in study design, data analysis, data interpretation, or writing of the report.

## AUTHOR CONTRIBUTIONS

Miriam Nakalembe, Megan J. Huchko, Jeffrey Martin: Conceptualization, data curation, methodology, project administration, formal analysis, funding acquisition, writing—review and editing. Philippa Makanga: Data curation, methodology, project administration, review and editing. Miriam Laker‐Oketta and Kambugu Andrew: Funding acquisition and Project administration.

## Data Availability

The data that support the findings of this study are available from the corresponding author upon reasonable request.
